# Supplementary material for: Unique Features of Extremely Halophilic Microbiota Inhabiting Solar Saltworks Fields of Vietnam
Source: Microorganisms. 2024 Sep 29;12(10):1975. doi: 10.3390/microorganisms12101975 (PMC11509607; doi:10.3390/microorganisms12101975)
Supplement: Supplementary file 1 [file microorganisms-12-01975-s001.zip › microorganisms-3184938-supplementary.pdf]

Table S1. Bacterial genera with ambiguous affiliations and relative abundances <1.0 %

Genus	VS7	VS5	VS2	VS9 sed	VS1new	HL345	VS1	VS4	VS6	HL450	VS3	VS8	VFS	VCR	STP	CP	SM16	SM19	SSR
Salinibacter	0.12	0	0.04	23.41	44.49	0.41	3.43	0.53	2.31	4.01	0	90.39	0.61	24.82	69.71	6.77	57.02	40.35	10.98
Salisaeta	0.43	2.84	0.35	0.79	18.35	0	13.83	3.36	51.36	0	12.53	0.22	15.53	4.76	2.24	28.99	0	0.38	19.21
Dactylococcopsis PCC-8305	0	3.16	0.10	0.12	0.43	0	9.02	3.71	12.47	0	13.42	0	11.50	0	0	15.23	0	0	5.32
Achromobacter	0.01	0	0	0	0	0	12.39	1.60	0	0	16.44	0	35.07	7.44	0.02	0	0	0	0
Unc. Bradymonadaceae	1.35	1.66	0.79	1.96	11.09	0	0	0.17	0	0	0	0	0	0	4.62	0	5.13	24.53	15.23
Halovibrio	0	0.21	0	0.80	0	39.90	0	0	0	21.39	0	0	0	0	0	0	2.39	0	0
Puniceicoccus	0.07	12.97	0.16	0.01	1.55	0	9.03	12.52	2.02	0	0	0	0	1.02	0.60	3.14	0	0	0
Halanaerobium	2.58	8.27	9.40	6.15	0	0.14	0.01	0	0.35	1.07	0	0	0	2.69	0.56	0	0	0	7.65
Unc. Halobacteroidaceae	0.02	0.17	0.23	0.92	1.19	0	0	0.18	0	0	0	0	0	0	0	35.11	0	0	0
Stenotrophomonas	0	0	0	0	0	0	8.48	1.18	0.03	0	5.66	0	15.08	6.20	0	0	0	0	0
Aquabacterium	0	0	0	0	0	0	1.69	0	0	0	7.78	0	18.90	5.52	0	0	0	0	0
Halomonas	0	0.94	0.18	0	0	16.29	0	0	0	6.28	0.43	0	0	2.35	0	0	0	0	0
Idiomarina	0.02	1.54	0.38	0	0	8.59	0	0	0.47	11.76	0.64	0	0	0	0	0	0	0	0
Rhodovibrio	1.50	0.09	0.88	3.16	2.01	1.38	2.05	0.24	10.02	0	0	0	0	0	0.67	0	0	0	0.45
Coraliomargarita	0	15.39	0.18	0	0	0	4.26	1.14	0.97	0	0	0	0	0.34	0	0	0	0	0
Aliifodinibius	2.53	0.67	1.17	0	0	7.30	0	0	0	6.15	0	1.51	0	0	0	0	0	2.72	0
Unc. Balneolaceae	2.96	1.35	0.35	0.01	0	0	0.33	1.17	1.50	0	8.85	1.16	0	0	0	0	1.31	1.77	0
Salinivibrio	0.10	0	0	0	0	0	0	0	0	0	19.58	0	0	0	0	0	0	0	0.44
Unc. Halomonadaceae	0.28	2.13	0.28	2.70	0	4.42	0	0	0	6.55	0	0	0	0	0	0	1.15	0.79	0.76
Psychrobacter	0	0	0	0	0	0	0	0	0	0	0	0	0	17.50	0	0	0	0	0
Unc. Phycisphaeraceae	6.45	0	0.58	2.10	0	0	0.02	0	0	0	0	0	0	0	0	0	6.41	0.24	1.23
Halofilum	0.46	1.04	0.20	0	0	6.04	0	0	0	6.55	0	0	0	0	0	0	1.42	0.65	0
Longimonas	0	0.81	0.13	0	1.49	0	1.02	0.08	4.86	0	3.20	0	2.34	0	0	0	0	0	2.28
Unc. Micavibrionaceae	0.25	0	0	0.42	1.14	3.77	0.88	2.01	0.69	5.88	0	0	0	0	0	0.19	0	0	0
Desulfovermiculus	0.57	2.10	3.55	6.39	0	0	0	0	0	0	0	0	0	0	0.31	0	1.32	0	0.73
Thiohalorhabdus	6.87	0.19	5.54	0.34	0	0	0	0	0	0	0	0	0	0	0	0	0.65	0	0
Psychroflexus	0	0.76	0.08	0	0.15	0	0.06	0	0	0	0	0	0	2.00	0.38	0	0	0	7.98
Truepera	3.08	2.37	0.40	0	0.62	0	0.94	2.06	0.83	0	0.37	0	0	0	0	0	0	0	0
Halopeptonella	0	1.12	0.42	0	1.00	2.09	0	3.02	0	0	0	0	0	2.89	0	0	0	0	0
Unc. E6aC02	0.54	1.03	1.39	1.28	0	0	0	0	0	0	0	0	0	0	0	0	0	0	5.28
Lentimonas	0	0.45	0.09	0.18	0	0	1.31	7.30	0	0	0	0	0	0	0	0	0	0	0
Unc. Marinilabiliaceae	2.47	0.58	3.62	0.39	0	0	0	0	0.21	0	0	0	0	0	0	0	0	0	1.97

Roseovarius	0	0	0	0	0	0	0	0	0	8.42	0	0	0	0	0.39	0	0	0	0
Unc. Rhodobacteraceae	2.36	0.02	0.25	0.21	0.01	0	2.81	2.99	0	0	0	0	0	0	0	0	0	0	0
Unc. Saprospiraceae	0.43	1.48	0.13	0	0	0	0.99	5.49	0.08	0	0	0	0	0	0	0	0	0	0
Roseivivax	0	0	0.65	0	2.12	0	1.11	2.16	0.27	0	0	0	0	0	1.13	0	0	0	0
WDS1C4	0	0	0	0	0.30	0	0	6.94	0	0	0	0	0	0	0	0	0	0	0
Verruc-01	0.17	0.33	1.13	0	0	0	1.80	1.21	1.03	0	0	0	0	0	0	0	0	0	1.44
Marinobacter	0.98	1.45	1.32	0	0	3.22	0	0	0.12	0	0	0	0	0	0	0	0	0	0
Halanaerobacter	0	0.25	0.16	1.29	0	0	0	0	0	0	0	0	0	0	0	4.73	0	0	0.55
Fusobacterium	0	0	0	0	0	0	0	0	0	0	0	0	0	6.01	0	0	0	0	0
Spirochaeta 2	0.56	0	1.77	0	0	0	0.10	0	0.10	0	0	0	0	0	0	0	0	0	3.12
Oceanicaulis	0.39	0	0.48	0	0.40	0	0.16	3.27	0.36	0	0.27	0	0	0	0.27	0	0	0	0
Gracilimonas	0.13	0.48	0.23	0	0.01	0	0.44	0.42	0	0	3.89	0	0	0	0	0	0	0	0
Ca. *Aquiluna*	0	3.36	0.12	0	0	0	0	1.69	0	0	0.22	0	0	0	0	0	0	0	0
Unc. C2U	1.43	0	0	0	0.41	0	1.79	0.59	0	0	0	0.51	0	0	0.60	0	0	0	0
Unc. Lenti-02	0	0.41	3.58	0.79	0	0	0.27	0.03	0	0	0	0	0	0	0	0	0	0	0
Maritimibacter	0	0	0	0	0	0	0	0	0	4.95	0	0	0	0	0	0	0	0	0
Halospina	0.57	2.00	0	0.17	0	0	0	0	0.38	0	0	0	0	0	0	0	0.46	0.22	0.99
Sphingomicrobium	0	0	0	0	0	0	0	0	0	4.68	0	0	0	0	0	0	0	0	0
Desulfosalsimonas	0.30	0	3.47	0.68	0	0	0	0	0	0	0	0	0	0	0	0	0	0	0
MAT-CR-P4-C12	0.10	0	0.24	0.86	0.04	0	0	0	0	0	0	0	0	0.05	0	0	0.83	2.17	0
Rhodopirellula	0.27	0.41	0.02	0	0	0	2.40	1.06	0	0	0	0	0	0	0.02	0	0	0	0
Limibaculum	0.90	0	0.79	0	0.68	0	0	1.81	0	0	0	0	0	0	0	0	0	0	0
Roseicyclus	0.35	0	0.23	0	0	0	1.22	2.14	0	0	0	0	0	0	0.11	0	0	0	0
Anaerobiospirillum	0	0	0	0	0	0	0	0	0	0	0	0	0	3.78	0	0	0	0	0
Guyparkeria	0	0.53	3.06	0	0	0	0.02	0	0	0	0	0	0	0	0	0	0	0	0
Wenzhouxiangella	2.81	0	0.34	0	0.41	0	0	0	0	0	0	0	0	0	0	0	0	0	0
Plesiomonas	0	0	0	0	0	0	0	0	0	0	0	0	0	3.53	0	0	0	0	0
Unc. Alcanivoracaceae	0.09	0	0	0.73	0	0	0	0	0	0	0	0	0	0	0	0	1.02	1.66	0
Peptoclostridium	0	0	0	0	0	0	0	0	0	0	0	0	0	3.40	0	0	0	0	0
DS001	0	2.64	0	0	0	0	0	0.54	0	0	0	0	0	0	0	0	0	0	0
Salinispirillum	0	1.07	0.03	0	0	1.97	0	0	0	0	0	0	0	0	0	0	0	0	0
Halobacillus	0	0	0	0	0	0	0	0	0	0	0	3.01	0	0	0	0	0	0	0
Albimonas	0.57	0	0.06	0	0	0	2.03	0.13	0.21	0	0	0	0	0	0	0	0	0	0
Unc. Hyphomonadaceae	0.13	0	0.23	0	0.32	0	1.54	0.49	0.20	0	0	0	0	0	0	0	0	0	0

SM1A02	0	0	0.16	0	0	0	0	0.04	0	0	0	0	0	0	0	2.70	0	0	0
Catalinimonas	0	0	0.12	0	0	0	0.20	0.21	0.68	0	1.64	0	0	0	0	0	0	0	0
Fusibacter	0.27	0.97	0.43	1.12	0	0	0	0	0	0	0	0	0	0	0	0	0	0	0
Unc. Rhodothermaceae	0.39	0	0	0.70	0.26	0	0.07	0	0	0	0.58	0	0	0	0	0	0	0.74	0
Pseudohongiella	0	1.44	0.15	0.46	0	0	0	0.17	0	0	0	0	0	0	0	0	0	0.33	0
Halanaerobaculum	0.01	0	0	0.01	0	0	0	0	0	0	0	0	0	0	0.06	2.45	0	0	0
Parvularcula	0	0	0	0	0.03	0	0.43	0.05	2.00	0	0	0	0	0	0	0	0	0	0
Owenweeksia	0.27	1.39	0.28	0.06	0	0	0	0.19	0.05	0	0	0	0	0	0	0	0	0	0.19
Legionella	0.49	0	0	0	0	0	0	0.60	0	1.34	0	0	0	0	0	0	0	0	0
Unc. Comamonadaceae	0	0	0	0	0	0	0	0	0	2.41	0	0	0	0	0	0	0	0	0
Desulfohalobium	0.17	0.26	0	1.95	0	0	0	0	0	0	0	0	0	0	0	0	0	0	0
Thiohalospira	0.42	0.66	0.29	0	0	0	0	0	0	0	0	0	0	0	0	0	0	0	0.93
Alcanivorax	0.05	0	0.46	1.68	0	0	0	0	0	0	0	0	0	0	0	0	0	0	0
Limimonas	0	0	0	0.34	0.59	0	0	0	0	0	0	0	0	0	1.23	0	0	0	0
Unc. Desulfosarcinaceae	0.01	0	1.64	0.00	0	0	0	0	0	0	0	0	0	0	0	0	0	0	0.32
Acinetobacter	0	0	0	0	0	0	0	0	0.93	0	0.64	0.42	0	0	0	0	0	0	0
Unc. Desulfobacteraceae	0.25	0.95	0	0.77	0	0	0	0	0	0	0	0	0	0	0	0	0	0	0
Cyanobium PCC-6307	0	0	0.48	0	0	0	0	0	0	1.47	0	0	0	0	0	0	0	0	0
Peredibacter	0.27	0.79	0.37	0	0	0	0	0.48	0	0	0	0	0	0	0	0	0	0	0
Qipengyuania	0	0	0	0	0	0	1.11	0.69	0	0	0	0	0	0	0	0	0	0	0
GKS98 freshwater group	0	0.15	0.04	0	0	0.65	0.39	0.50	0	0	0	0	0	0	0	0	0	0	0
Pontimonas	0	1.36	0	0	0	0	0	0	0.30	0	0	0	0	0	0	0	0	0	0
Methyloversatilis	0	0	0	0	0	0	0	0	0	0	1.64	0	0	0	0	0	0	0	0
Flexistipes	0.28	0	1.16	0.19	0	0	0	0	0	0	0	0	0	0	0	0	0	0	0
Silicimonas	1.58	0	0.04	0	0	0	0	0	0	0	0	0	0	0	0	0	0	0	0
Cnuella	0	0	0	0	0	0	0	0	0	1.60	0	0	0	0	0	0	0	0	0
Unc. Desulfobulbaceae	1.34	0	0.24	0	0	0	0.02	0	0	0	0	0	0	0	0	0	0	0	0
Sediminimonas	1.59	0	0	0	0	0	0	0	0	0	0	0	0	0	0	0	0	0	0
Unc. Bacillaceae	0	0.34	0	0	0	0	0	0.11	0	0	0	1.07	0	0	0	0	0	0	0
Unc. Ectothiorhodospiraceae	0	0	0.09	0	0	0	0	0.23	0	0	0	0	0	0	0	0	0.57	0.61	0
Phormidium MBIC10003	0	0	0	0	0	0	0	0	0	0	1.48	0	0	0	0	0	0	0	0
Unc. Methylomonadaceae	0	0	0	0	0	0	0	0	0	1.47	0	0	0	0	0	0	0	0	0
Halospirulina CCC Baja-95 Cl.2	0.01	0	1.21	0	0	0	0	0.01	0.22	0	0	0	0	0	0	0	0	0	0
Tangfeifania	0	0	1.39	0.01	0	0	0	0	0	0	0	0	0	0	0	0	0	0	0

Halodurantibacterium	0	0	0	0	0	0	0	0	0	0	0	0	0	0	1.38	0	0	0	0
Ca. *Endoecteinascidia*	0	0	0	0	0	0	0	1.36	0	0	0	0	0	0	0	0	0	0	0
Unc. Cyclobacteriaceae	0.47	0.15	0.10	0	0	0	0	0	0	0	0	0	0	0	0	0	0	0	0.61
ADurb.Bin120	1.05	0	0.26	0	0	0	0	0	0	0	0	0	0	0	0	0	0	0	0
Sediminispirochaeta	0	0	1.29	0	0	0	0	0	0	0	0	0	0	0	0	0	0	0	0
Bacteroides	0	0	0	0	0	0	0	0	0	0	0	0	0	1.28	0	0	0	0	0
Sumerlaea	0	0	0.78	0	0	0	0	0	0	0	0	0	0	0	0	0	0	0	0.49
Litoricola	0	0.36	0	0	0	0	0.16	0.59	0.15	0	0	0	0	0	0	0	0	0	0
Methylosphaera	0	0	0	0	0	1.23	0	0	0	0	0	0	0	0	0	0	0	0	0
Anaerophaga	0.21	0.16	0.86	0	0	0	0	0	0	0	0	0	0	0	0	0	0	0	0
Phycisphaera	0	0	0	0	0	0	0.56	0.63	0	0	0	0	0	0	0	0	0	0	0
Geotoga	0	0.65	0.52	0	0	0	0	0	0	0	0	0	0	0	0	0	0	0	0
Izimaplasma	0	0.53	0.13	0	0	0.09	0	0.33	0	0	0	0	0	0	0.09	0	0	0	0
Geitlerinema PCC-7105	0	0	1.14	0	0	0	0	0	0	0	0	0	0	0	0	0	0	0	0
Rhodobaculum	0.27	0	0	0	0	0	0.85	0	0	0	0	0	0	0	0	0	0	0	0
Rhodovulum	0	0	0.06	0	0	0	0.68	0.31	0.07	0	0	0	0	0	0	0	0	0	0
Arsenophonus	0	0	0	0	1.11	0	0	0	0	0	0	0	0	0	0	0	0	0	0
Ca. *Berkiella*	0.96	0	0	0	0	0	0	0.15	0	0	0	0	0	0	0	0	0	0	0
Aquisalimonas	0	0	0	0	0	1.10	0	0	0	0	0	0	0	0	0	0	0	0	0
Coxiella	0	0	0	0	0	0	0	0.40	0	0	0	0	0	0	0	0	0.67	0	0
Ferrimonas	0	0	0	1.05	0	0	0	0	0	0	0	0	0	0	0	0	0	0	0
Arhodomonas	1.05	0	0	0	0	0	0	0	0	0	0	0	0	0	0	0	0	0	0
Desulfotignum	0	0	1.01	0	0	0	0	0	0	0	0	0	0	0	0	0	0	0	0
HIMB11	0	0	0.03	0	0	0	0.27	0.71	0	0	0	0	0	0	0	0	0	0	0
Unc. Methylohalomonas	0.86	0	0	0	0	0.13	0	0	0	0	0	0	0	0	0	0	0	0	0
Unc. Arenicellaceae	0	0.12	0	0	0	0	0.10	0.67	0.10	0	0	0	0	0	0	0	0	0	0
Thiohalobacter	0.97	0	0	0	0	0	0	0	0	0	0	0	0	0	0	0	0	0	0
Silanimonas	0	0	0	0	0	0	0	0	0	0	0	0	0.96	0	0	0	0	0	0
Haloferula	0	0	0.23	0	0	0	0	0.73	0	0	0	0	0	0	0	0	0	0	0
Geitlerinema PCC-9228	0	0.22	0	0.70	0	0	0	0	0	0	0	0	0	0	0	0	0	0	0.03
Unc. Halorhodospiraceae	0.41	0	0	0	0	0	0	0	0	0	0	0	0	0	0	0	0	0	0.51
OM27 clade	0.41	0	0	0	0	0	0	0.49	0	0	0	0	0	0	0	0	0	0	0
Thiohalophilus	0.90	0	0	0	0	0	0	0	0	0	0	0	0	0	0	0	0	0	0
Algisphaera	0	0	0	0	0	0	0.37	0	0	0	0	0	0	0	0	0	0	0	0.53

Mameliella	0	0	0	0	0	0	0	0.88	0	0	0	0	0	0	0	0	0	0	0
Unc. Prolixibacteraceae	0.26	0	0.59	0	0	0	0	0	0	0	0	0	0	0	0	0	0	0	0
Marispirillum	0.43	0	0.36	0	0	0	0	0	0.05	0	0	0	0	0	0	0	0	0	0
Tropicimonas	0	0	0.42	0	0	0	0.39	0	0	0	0	0	0	0	0	0	0	0	0
Phaeodactylibacter	0.39	0.20	0	0	0	0	0	0.19	0	0	0	0	0	0	0	0	0	0	0
Fodinibius	0	0	0	0	0	0	0	0	0	0	0	0	0	0	0	0	0	0.78	0
Unc. Nitriliruptoraceae	0.54	0	0	0	0	0	0	0.24	0	0	0	0	0	0	0	0	0	0	0
Salicola	0	0	0	0.76	0	0	0	0	0	0	0	0	0	0	0	0	0	0	0
Unc. Rhodospirillaceae	0.23	0	0	0.51	0	0	0	0	0	0	0	0	0	0	0	0	0	0	0
Salinarimonas	0.66	0	0.08	0	0	0	0	0	0	0	0	0	0	0	0	0	0	0	0
Halocella	0	0.19	0.37	0.17	0	0	0	0	0	0	0	0	0	0	0	0	0	0	0
Nioella	0	0	0	0	0	0	0.72	0	0	0	0	0	0	0	0	0	0	0	0
Unc. Francisellaceae	0.42	0	0	0.28	0	0	0	0	0	0	0	0	0	0	0	0	0	0	0
Unc. Thioprofundaceae	0	0	0.23	0	0	0	0	0.46	0	0	0	0	0	0	0	0	0	0	0
Aquichromatium	0	0	0	0	0	0	0	0	0	0	0	0	0	0	0	0.68	0	0	0
Pseudohaliea	0	0	0	0	0	0	0	0.68	0	0	0	0	0	0	0	0	0	0	0
Unc. Rickettsiaceae	0	0	0	0	0.40	0	0	0.28	0	0	0	0	0	0	0	0	0	0	0
Bdellovibrio	0.10	0	0	0	0	0	0	0.57	0	0	0	0	0	0	0	0	0	0	0
Desulfovibrio	0	0	0.25	0.40	0	0	0	0	0	0	0	0	0	0	0	0	0	0	0
Alkalispirochaeta	0	0	0	0	0	0	0.53	0.08	0	0	0	0	0	0	0	0	0	0	0
Woeseia	0.21	0	0.38	0	0	0	0	0	0	0	0	0	0	0	0	0	0	0	0
Sporohalobacter	0	0	0	0.52	0	0	0	0	0	0	0	0	0	0	0.07	0	0	0	0
Synechococcus MBIC10613	0	0	0.59	0	0	0	0	0	0	0	0	0	0	0	0	0	0	0	0
Unc. Vermiphilaceae	0.38	0	0	0	0	0	0	0.20	0	0	0	0	0	0	0	0	0	0	0
Unc. SM2D12	0	0	0	0	0	0	0	0.57	0	0	0	0	0	0	0	0	0	0	0
Unc. Criblamydiaceae	0.57	0	0	0	0	0	0	0	0	0	0	0	0	0	0	0	0	0	0
Unc. SB-5	0	0.12	0.37	0.03	0	0	0	0	0	0	0	0	0	0	0	0	0	0	0.05
Luminiphilus	0	0	0	0	0	0	0	0.53	0	0	0	0	0	0	0	0	0	0	0
Unc. Holosporaceae	0	0	0	0	0	0	0	0	0	0.53	0	0	0	0	0	0	0	0	0
Unc. NS9 marine group	0	0	0	0	0	0	0	0.53	0	0	0	0	0	0	0	0	0	0	0
Unc. Pseudohongiellaceae	0.22	0.27	0	0	0	0	0	0	0	0	0	0	0	0	0	0	0	0	0
Halarsenatibacter	0	0	0.23	0.26	0	0	0	0	0	0	0	0	0	0	0	0	0	0	0
Desulfopila	0	0	0.47	0	0	0	0	0	0	0	0	0	0	0	0	0	0	0	0
OM60(NOR5) clade	0	0	0	0	0	0	0	0.47	0	0	0	0	0	0	0	0	0	0	0

TX1A-55	0	0	0	0	0	0	0	0	0.45	0	0	0	0	0	0	0	0	0	0
JTB255 marine benthic group	0	0	0.45	0	0	0	0	0	0	0	0	0	0	0	0	0	0	0	0
Unc. Desulfococcaceae	0	0	0.45	0	0	0	0	0	0	0	0	0	0	0	0	0	0	0	0
Sagittula	0	0	0	0	0	0	0.44	0	0	0	0	0	0	0	0	0	0	0	0
Unc. AKAU3564 sediment group	0	0	0.44	0	0	0	0	0	0	0	0	0	0	0	0	0	0	0	0
Unc. Methylacidiphilaceae	0	0	0.43	0	0	0	0	0	0	0	0	0	0	0	0	0	0	0	0
Balneola	0	0	0	0	0	0	0	0.43	0	0	0	0	0	0	0	0	0	0	0
Unc. Pirellulaceae	0	0	0.26	0.07	0	0	0.09	0	0	0	0	0	0	0	0	0	0	0	0
Unc. Thiohalobacteraceae	0.41	0	0	0	0	0	0	0	0	0	0	0	0	0	0	0	0	0	0
Citreimonas	0	0	0.41	0	0	0	0	0	0	0	0	0	0	0	0	0	0	0	0
Unc. Paracaedibacteraceae	0	0	0	0	0	0	0	0.41	0	0	0	0	0	0	0	0	0	0	0
Unc. Rhodobiaceae	0	0	0.40	0	0	0	0	0	0	0	0	0	0	0	0	0	0	0	0
SBZC-1223	0	0	0.40	0	0	0	0	0	0	0	0	0	0	0	0	0	0	0	0
Thermoflavifilum	0.40	0	0	0	0	0	0	0	0	0	0	0	0	0	0	0	0	0	0
Algoriphagus	0	0.32	0.07	0	0	0	0	0	0	0	0	0	0	0	0	0	0	0	0
Unc. Bacteroidetes BD2-2	0	0	0.39	0	0	0	0	0	0	0	0	0	0	0	0	0	0	0	0
Sediminibacterium	0.02	0	0	0	0	0	0.11	0.03	0	0	0.23	0	0	0	0	0	0	0	0
Unc. DEV007	0.38	0	0	0	0	0	0	0	0	0	0	0	0	0	0	0	0	0	0
Sphingomonas	0.25	0	0	0	0	0	0.09	0.03	0	0	0	0	0	0	0	0	0	0	0
Unc. Spirochaetaceae	0	0	0.37	0	0	0	0	0	0	0	0	0	0	0	0	0	0	0	0
Unc. Oligoflexaceae	0	0	0	0	0	0	0	0.37	0	0	0	0	0	0	0	0	0	0	0
Spirochaeta	0	0	0.36	0	0	0	0	0	0	0	0	0	0	0	0	0	0	0	0
Unc. Babeliaceae	0.16	0	0	0.19	0	0	0	0	0	0	0	0	0	0	0	0	0	0	0
Unc. Hydrogenedensaceae	0.08	0	0.27	0	0	0	0	0	0	0	0	0	0	0	0	0	0	0	0
Tranquillimonas	0.16	0	0.19	0	0	0	0	0	0	0	0	0	0	0	0	0	0	0	0
Unc. Izemoplasmataceae	0	0	0.34	0	0	0	0	0	0	0	0	0	0	0	0	0	0	0	0
Geoalkalibacter	0	0	0.33	0	0	0	0	0	0	0	0	0	0	0	0	0	0	0	0
Unc. Lentimicrobiaceae	0	0	0.33	0	0	0	0	0	0	0	0	0	0	0	0	0	0	0	0
Unc. Lactobacillaceae	0	0	0	0	0	0	0	0	0	0	0	0	0	0	0.33	0	0	0	0
Caedibacter	0	0	0	0	0	0	0.29	0.04	0	0	0	0	0	0	0	0	0	0	0
Ca. Marispirochaeta	0	0	0.31	0	0	0	0	0	0	0	0	0	0	0	0	0	0	0	0
MSBL7	0	0	0.31	0	0	0	0	0	0	0	0	0	0	0	0	0	0	0	0
Natroniella	0.30	0	0	0	0	0	0	0	0	0	0	0	0	0	0	0	0	0	0
Thiohalospira	0.30	0	0	0	0	0	0	0	0	0	0	0	0	0	0	0	0	0	0

Unc. Melioribacteraceae	0	0	0.26	0.04	0	0	0	0	0	0	0	0	0	0	0	0	0	0	0
Caenispirillum	0	0	0.29	0	0	0	0	0	0	0	0	0	0	0	0	0	0	0	0
Melioribacter	0	0	0.29	0	0	0	0	0	0	0	0	0	0	0	0	0	0	0	0
Dichotomicrobium	0.29	0	0	0	0	0	0	0	0	0	0	0	0	0	0	0	0	0	0
SC103	0	0	0.05	0.24	0	0	0	0	0	0	0	0	0	0	0	0	0	0	0
Thioalkalispira	0	0	0.29	0	0	0	0	0	0	0	0	0	0	0	0	0	0	0	0
Desulfonatronobacter	0	0	0.29	0	0	0	0	0	0	0	0	0	0	0	0	0	0	0	0
Unc. Anaerolineaceae	0	0	0.27	0	0	0	0	0	0	0	0	0	0	0	0	0	0	0	0
Halovulum	0.27	0	0	0	0	0	0	0	0	0	0	0	0	0	0	0	0	0	0
Orenia	0.16	0	0.11	0	0	0	0	0	0	0	0	0	0	0	0	0	0	0	0
Unc. SBXY-748	0.02	0	0.23	0	0	0	0	0	0	0	0	0	0	0	0	0	0	0	0
Geothermobacter	0	0	0.25	0	0	0	0	0	0	0	0	0	0	0	0	0	0	0	0
Lactobacillus	0	0	0	0	0	0	0	0	0	0	0.25	0	0	0	0	0	0	0	0
Rubinisphaera	0.25	0	0	0	0	0	0	0	0	0	0	0	0	0	0	0	0	0	0
Thiomicrorhabdus	0	0	0.24	0	0	0	0	0	0	0	0	0	0	0	0	0	0	0	0
Cerasicoccus	0	0	0	0	0	0	0.11	0.13	0	0	0	0	0	0	0	0	0	0	0
C1-B045	0.24	0	0	0	0	0	0	0	0	0	0	0	0	0	0	0	0	0	0
Simkania	0.24	0	0	0	0	0	0	0	0	0	0	0	0	0	0	0	0	0	0
Unc. Cryomorphaceae	0	0.24	0	0	0	0	0	0	0	0	0	0	0	0	0	0	0	0	0
Algivirga	0	0	0	0	0	0	0	0	0.24	0	0	0	0	0	0	0	0	0	0
Anderseniella	0.23	0	0	0	0	0	0	0	0	0	0	0	0	0	0	0	0	0	0
MSBL3	0	0	0.22	0	0	0	0	0	0	0	0	0	0	0	0	0	0	0	0
Thioprofundum	0	0	0.22	0	0	0	0	0	0	0	0	0	0	0	0	0	0	0	0
Unc. UBA12409	0	0	0	0.22	0	0	0	0	0	0	0	0	0	0	0	0	0	0	0
Robiginitalea	0.22	0	0	0	0	0	0	0	0	0	0	0	0	0	0	0	0	0	0
Chthonobacter	0	0	0.21	0	0	0	0	0	0	0	0	0	0	0	0	0	0	0	0
Unc. Terasakiellaceae	0	0	0.11	0	0	0	0	0.09	0	0	0	0	0	0	0	0	0	0	0
Unc. Chromatiaceae	0	0	0	0	0	0	0	0.19	0	0	0	0	0	0	0	0	0	0	0
Francisella	0	0	0	0	0	0.19	0	0	0	0	0	0	0	0	0	0	0	0	0
Henriciella	0.18	0	0	0	0	0	0	0	0	0	0	0	0	0	0	0	0	0	0
Paracoccus	0	0	0	0	0	0	0	0	0	0	0	0	0	0	0.18	0	0	0	0
Unc. Kiloniellaceae	0.16	0	0	0	0	0	0	0	0	0	0	0	0	0	0	0	0	0	0
Ilumatobacter	0	0	0.16	0	0	0	0	0	0	0	0	0	0	0	0	0	0	0	0
Unc. Chitinispirillum	0	0	0.16	0	0	0	0	0	0	0	0	0	0	0	0	0	0	0	0

RCP1-48	0	0	0.16	0	0	0	0	0	0	0	0	0	0	0	0	0	0	0	0
Sulfurovum	0	0	0.15	0	0	0	0	0	0	0	0	0	0	0	0	0	0	0	0
Oceanotoga	0	0	0	0.15	0	0	0	0	0	0	0	0	0	0	0	0	0	0	0
Roseomonas	0	0	0.15	0	0	0	0	0	0	0	0	0	0	0	0	0	0	0	0
Aquicella	0.14	0	0	0	0	0	0	0	0	0	0	0	0	0	0	0	0	0	0
Fuchsiella	0	0	0	0.14	0	0	0	0	0	0	0	0	0	0	0	0	0	0	0
Unc. Nodosilineaceae	0	0	0.14	0	0	0	0	0	0	0	0	0	0	0	0	0	0	0	0
Unc. Petrotogaceae	0	0	0	0.14	0	0	0	0	0	0	0	0	0	0	0	0	0	0	0
SCGC AAA164-E04	0	0	0	0	0	0	0	0.14	0	0	0	0	0	0	0	0	0	0	0
Unc. Deferribacteraceae	0	0	0.14	0	0	0	0	0	0	0	0	0	0	0	0	0	0	0	0
Gimesia	0.10	0.03	0	0	0	0	0	0	0	0	0	0	0	0	0	0	0	0	0
Thiosocius	0.13	0	0	0	0	0	0	0	0	0	0	0	0	0	0	0	0	0	0
Unc. ST-12K33	0	0	0.13	0	0	0	0	0	0	0	0	0	0	0	0	0	0	0	0
Thiomicrospira	0	0	0.13	0	0	0	0	0	0	0	0	0	0	0	0	0	0	0	0
Ellin6055	0.13	0	0	0	0	0	0	0	0	0	0	0	0	0	0	0	0	0	0
Unc. Arcobacteraceae	0.13	0	0	0	0	0	0	0	0	0	0	0	0	0	0	0	0	0	0
Burkholderia	0	0	0	0	0	0	0.13	0	0	0	0	0	0	0	0	0	0	0	0
Pirellula	0.07	0	0	0	0	0	0	0.06	0	0	0	0	0	0	0	0	0	0	0
Unc. OM182 clade	0.12	0	0	0	0	0	0	0	0	0	0	0	0	0	0	0	0	0	0
Unc. Mitochondria	0.03	0	0	0	0	0	0	0.07	0.03	0	0	0	0	0	0	0	0	0	0
Faucicola	0	0	0	0	0	0	0	0	0	0	0.12	0	0	0	0	0	0	0	0
Unc. AB1	0	0	0	0	0	0	0.12	0	0	0	0	0	0	0	0	0	0	0	0
Microvirga	0	0	0	0	0	0	0	0	0.12	0	0	0	0	0	0	0	0	0	0
Unc. Halanaerobiaceae	0	0	0.12	0	0	0	0	0	0	0	0	0	0	0	0	0	0	0	0
Marinobacterium	0	0.07	0.04	0	0	0	0	0	0	0	0	0	0	0	0	0	0	0	0
Unc. Azospirillaceae	0.11	0	0	0	0	0	0	0	0	0	0	0	0	0	0	0	0	0	0
Unc. Holophagaceae	0	0	0	0	0	0	0	0.03	0	0	0.08	0	0	0	0	0	0	0	0
Membranicola	0.11	0	0	0	0	0	0	0	0	0	0	0	0	0	0	0	0	0	0
Longimonas	0	0	0	0.11	0	0	0	0	0	0	0	0	0	0	0	0	0	0	0
Bryobacter	0	0	0.11	0	0	0	0	0	0	0	0	0	0	0	0	0	0	0	0
Unc. Crocinitomicaceae	0	0	0	0	0	0	0	0.10	0	0	0	0	0	0	0	0	0	0	0
Planctopirus	0	0	0.10	0	0	0	0	0	0	0	0	0	0	0	0	0	0	0	0
Desulfocella	0	0	0.10	0	0	0	0	0	0	0	0	0	0	0	0	0	0	0	0
Unc. Calditrichaceae	0	0	0.099	0	0	0	0	0	0	0	0	0	0	0	0	0	0	0	0

Unc. Cellvibrionaceae	0.095	0	0	0	0	0	0	0	0	0	0	0	0	0	0	0	0	0	0
Tistlia	0	0	0	0	0	0	0	0.092	0	0	0	0	0	0	0	0	0	0	0
Unc. LWQ8	0.091	0	0	0	0	0	0	0	0	0	0	0	0	0	0	0	0	0	0
Unc. B18	0	0	0	0.091	0	0	0	0	0	0	0	0	0	0	0	0	0	0	0
Jannaschia	0	0	0.090	0	0	0	0	0	0	0	0	0	0	0	0	0	0	0	0
Lacimicrobium	0	0	0.025	0	0	0	0	0.064	0	0	0	0	0	0	0	0	0	0	0
Unc. Magnetospiraceae	0	0	0.088	0	0	0	0	0	0	0	0	0	0	0	0	0	0	0	0
Unc. JG30-KF-CM45	0	0	0	0	0	0	0	0	0.082	0	0	0	0	0	0	0	0	0	0
Thioalkalivibrio	0.082	0	0	0	0	0	0	0	0	0	0	0	0	0	0	0	0	0	0
Unc. Simkaniaceae	0.078	0	0	0	0	0	0	0	0	0	0	0	0	0	0	0	0	0	0
Unc. Leptospiraceae	0	0	0.078	0	0	0	0	0	0	0	0	0	0	0	0	0	0	0	0
Marivirga	0	0	0	0	0	0	0	0	0	0	0.071	0	0	0	0	0	0	0	0
Acholeplasma	0	0	0.071	0	0	0	0	0	0	0	0	0	0	0	0	0	0	0	0
Unc. Acetobacteraceae	0	0	0.067	0	0	0	0	0	0	0	0	0	0	0	0	0	0	0	0
OLB12	0.065	0	0	0	0	0	0	0	0	0	0	0	0	0	0	0	0	0	0
Unc. Gimesiaceae	0.065	0	0	0	0	0	0	0	0	0	0	0	0	0	0	0	0	0	0
Oscillatoria PCC-6304	0	0	0.065	0	0	0	0	0	0	0	0	0	0	0	0	0	0	0	0
Ca. *Omnitrophus*	0	0	0	0.064	0	0	0	0	0	0	0	0	0	0	0	0	0	0	0
Unc. Thiomicrospiraceae	0	0	0.059	0	0	0	0	0	0	0	0	0	0	0	0	0	0	0	0
Salinivirga	0.059	0	0	0	0	0	0	0	0	0	0	0	0	0	0	0	0	0	0
Unc. Flavobacteriaceae	0	0	0	0	0	0.058	0	0	0	0	0	0	0	0	0	0	0	0	0
Anaerolineaceae UCG-001	0	0	0.057	0	0	0	0	0	0	0	0	0	0	0	0	0	0	0	0
Marinilabilia	0	0	0.057	0	0	0	0	0	0	0	0	0	0	0	0	0	0	0	0
Unc. MSB-3C8	0	0	0.057	0	0	0	0	0	0	0	0	0	0	0	0	0	0	0	0
Pseudenhygromyxa	0.055	0	0	0	0	0	0	0	0	0	0	0	0	0	0	0	0	0	0
Cryomorpha	0	0	0	0	0	0	0	0.053	0	0	0	0	0	0	0	0	0	0	0
Natronotalea	0	0	0	0.005	0.047	0	0	0	0	0	0	0	0	0	0	0	0	0	0
Unc. SG8-4	0	0	0.050	0	0	0	0	0	0	0	0	0	0	0	0	0	0	0	0
Nitrospira	0.049	0	0	0	0	0	0	0	0	0	0	0	0	0	0	0	0	0	0
Bermanella	0	0	0	0	0	0	0	0.048	0	0	0	0	0	0	0	0	0	0	0
Unc. Syntrophobacteraceae	0	0	0	0.047	0	0	0	0	0	0	0	0	0	0	0	0	0	0	0
YC-ZSS-LKJ63	0	0	0.046	0	0	0	0	0	0	0	0	0	0	0	0	0	0	0	0
Unc. Halieaceae	0	0	0.046	0	0	0	0	0	0	0	0	0	0	0	0	0	0	0	0
Salinisphaera	0.046	0	0	0	0	0	0	0	0	0	0	0	0	0	0	0	0	0	0

Halonatronum	0	0	0	0.044	0	0	0	0	0	0	0	0	0	0	0	0	0	0	0
Unc. Ferrimonadaceae	0	0	0	0.044	0	0	0	0	0	0	0	0	0	0	0	0	0	0	0
Levilinea	0	0	0.044	0	0	0	0	0	0	0	0	0	0	0	0	0	0	0	0
Methylophaga	0	0	0.042	0	0	0	0	0	0	0	0	0	0	0	0	0	0	0	0
Gramella	0	0	0.038	0	0	0	0	0	0	0	0	0	0	0	0	0	0	0	0
Flexithrix	0	0	0	0	0	0	0	0	0.036	0	0	0	0	0	0	0	0	0	0
Unc. Salinibacteraceae	0	0	0	0.030	0.004	0	0	0	0	0	0	0	0	0	0	0	0	0	0
Unc. Christensenellaceae	0	0	0.032	0	0	0	0	0	0	0	0	0	0	0	0	0	0	0	0
Unc. Idiomarinaceae	0	0	0	0	0	0.029	0	0	0	0	0	0	0	0	0	0	0	0	0
Sandaracinus	0	0	0	0	0	0	0	0.028	0	0	0	0	0	0	0	0	0	0	0
Unc. BIrii41	0	0	0	0	0	0	0	0	0.027	0	0	0	0	0	0	0	0	0	0
Unc. EC3	0.026	0	0	0	0	0	0	0	0	0	0	0	0	0	0	0	0	0	0
Unc. ML635J-40 aquatic group	0	0	0.025	0	0	0	0	0	0	0	0	0	0	0	0	0	0	0	0
Maritalea	0.020	0	0	0	0	0	0	0	0	0	0	0	0	0	0	0	0	0	0
Desulfitibacter	0	0	0	0.017	0	0	0	0	0	0	0	0	0	0	0	0	0	0	0
Unc. Paracoccaceae	0	0	0	0	0	0	0	0.017	0	0	0	0	0	0	0	0	0	0	0
Hoppeia	0.016	0	0	0	0	0	0	0	0	0	0	0	0	0	0	0	0	0	0
Adhaeribacter	0	0	0	0	0	0	0	0	0	0	0	0	0	0	0.016	0	0	0	0
Unc. Hungateiclostridiaceae	0	0	0.015	0	0	0	0	0	0	0	0	0	0	0	0	0	0	0	0
Unc. Moduliflexaceae	0	0	0.015	0	0	0	0	0	0	0	0	0	0	0	0	0	0	0	0
Unc. Rubidibacteraceae	0	0	0	0	0	0	0.014	0	0	0	0	0	0	0	0	0	0	0	0
Ca. *Desulfamplus*	0.013	0	0	0	0	0	0	0	0	0	0	0	0	0	0	0	0	0	0
Unc. Desulfarculaceae	0	0	0.008	0	0	0	0	0	0	0	0	0	0	0	0	0	0	0	0
Salinihabitans	0.007	0	0	0	0	0	0	0	0	0	0	0	0	0	0	0	0	0	0
Unc. Methylococcaceae	0.007	0	0	0	0	0	0	0	0	0	0	0	0	0	0	0	0	0	0
Malaciobacter	0	0	0.006	0	0	0	0	0	0	0	0	0	0	0	0	0	0	0	0
Aestuariivita	0	0	0	0	0	0	0	0.006	0	0	0	0	0	0	0	0	0	0	0
Unc. 37-13	0	0	0	0	0	0	0	0.006	0	0	0	0	0	0	0	0	0	0	0
Unc. Desulfonatronovibrionaceae	0	0	0	0.005	0	0	0	0	0	0	0	0	0	0	0	0	0	0	0
Galenea	0	0	0.004	0	0	0	0	0	0	0	0	0	0	0	0	0	0	0	0
Unc. Thioalkalibacteraceae	0	0	0.004	0	0	0	0	0	0	0	0	0	0	0	0	0	0	0	0
Selenihalanaerobacter	0	0	0	0.002	0	0	0	0	0	0	0	0	0	0	0	0	0	0	0
Unaffiliated 0319-6G20	0.03	0	0.00	0	0	0	0	0	0	0	0	0	0	0	0	0	0	0	0
Unaffiliated 113B434	0	0	0.07	0	0	0	0	0	0	0	0	0	0	0	0	0	0	0	0

Unaffiliated Absconditabacteriales (SR1)	0.01	0.12	0.46	0	0	0	0	0	0	0	0	0	0	0	0	0	0	0	0
Unaffiliated ABY1	0	0.01	0	0.20	0	0	0	0	0	0	0	0	0	0	0	0	0	0	0
Unaffiliated Acetothermiia	2.20	1.17	1.71	1.22	0	0	0	0	0	0	0	0	0	0	0	0	0	0	2.45
Unaffiliated Alphaproteobacteria	0.68	0	0	0.08	0.83	0	2.08	1.39	0.17	0	0	1.66	0	0	1.80	0	0	0	0
Unaffiliated Babeliales	0.26	0.71	0	0.13	0	0	0.18	1.28	0	0	0	0	0	0	0	0	0	0	0
Unaffiliated Bacillota	0	0.01	0.20	0.17	0	0	0	0	0	0	0	0	0	0	0	0	1.74	0.59	0
Unaffiliated Bacteriovoracales	0.05	0	0	0	0	0	0	0	0	0	0	0	0	0	0	0	0	0	0
Unaffiliated Bacteroidales	0	0	0.06	0	0	0	0	0	0	0	0	0	0	0	0	0	0	0	0
Unaffiliated Bacteroidia	0.11	0	0.05	0.00	0	0	0	0	0	0	0	0	0	0	0	0	0	1.89	0
Unaffiliated BD2-11 terrestrial group	0	0.49	1.42	0	0	0	0	0	0	0	0	0	0	1.00	0	0	0	0	0
Unaffiliated Bradymonadales	0.65	0	0.01	0.01	0	0	0.10	0.18	0.06	0	0	0	0	0	0	0	0	0	0
Unaffiliated Brocadiae	0	0	0.07	0	0	0	0	0	0	0	0	0	0	0	0	0	0	0	0
Unaffiliated Ca. Campbellbacteria	0	0.13	0	0	0	0	0	0.61	0.05	0	0	0	0	0	0	0	0	0	0
Unaffiliated Ca. Collierbacteria	0.25	0	0	0	0	0	0	0	0	0	0	0	0	0	0	0	0	0	0
Unaffiliated Ca. Falkowbacteria	0	0.44	0	0	0	0	0	0	0	0	0	0	0	0	0	0	0	0	0
Unaffiliated Ca. Kaiserbacteria	0.25	0.86	0.05	0	0	0	0	0	0	0	0	0	0	0	0	0	0	0	0
Unaffiliated Ca. Kerfeldbacteria	0.59	0	0	0	0	0	0	0	0	0	0	0	0	0	0	0	0	0	0
Unaffiliated Ca. Pacebacteria	0.34	0	0	0	0	0	0	0	0	0	0	0	0	0	0	0	0	0	0
Unaffiliated Ca. Uhrbacteria	1.75	0	0	0	0	0	0	0	0	0	0	0	0	0	0	0	0	0	0
Unaffiliated Chitinophagales	0.10	0	0.14	7.54	4.70	0.35	0.29	1.54	0	0	0	0	0	0	12.36	0	9.49	9.25	0
Unaffiliated Chlamydiales	0.68	0	0	0	0	0	0	0	0	0	0	0	0	0	0	0	0	0	0
Unaffiliated Chloroplast	0	1.73	0.14	0	0	0	0.06	0.35	0.30	0	0	0	0	0	0	0	0	0	0
Unaffiliated Chromatiales	0.83	0	0	0	0	0	0	0.02	0	0	0	0	0	0	0	0	0	0	0
Unaffiliated Clostridia vadinBB60 group	0	0	0.03	0.01	0	0	0	0	0	0	0	0	0	0	0	0	0	0	0
Unaffiliated CPR2	0.04	0	0	0	0	0	0	0	0	0	0	0	0	0	0	0	0	0	0
Unaffiliated Cyanobacteriales	0	0	0.00	0.00	0	0	0	0	0	0	0	0	0	0	0	0	0	0	0
Unaffiliated Cytophagales	0	0.45	0	0.08	0	0	1.81	0.43	1.02	0	0	0	0	0	0	0	1.71	0	0.86
Unaffiliated Dadabacteriales	0.17	0	0.04	0	0	0	0	0	0	0	0	0	0	0	0	0	0	0	0
Unaffiliated Deferribacterales	0.03	0	0.36	0	0	0	0	0	0	0	0	0	0	0	0	0	0	0	0
Unaffiliated Desulfitobacteriales	0.18	0	0	0	0	0	0	0	0	0	0	0	0	0	0	0	0	0	0
Unaffiliated Desulfobacterales	0.01	0	0	0	0	0	0	0	0	0	0	0	0	0	0	0	0	0	0
Unaffiliated Desulfobacterota	0	0	0	0.02	0	0	0	0.04	0	0	0	0	0	0	0	0	0	0	0
Unaffiliated Desulfovibrionales	0	0.01	0.10	0.04	0	0	0	0	0	0	0	0	0	0	0	0	0	0	0
Unaffiliated Desulfuromonadales	0	0	0.64	0	0	0	0	0	0	0	0	0	0	0	0	0	0	0	0

Unaffiliated Dissulfuribacterales	0	0	0.03	0	0	0	0	0	0	0	0	0	0	0	0	0	0	0	0
Unaffiliated Dojkabacteria	0.92	0	0.02	0	0	0	0	0	0	0	0	0	0	0	0	0	0	0	0
Unaffiliated EC3	0.03	0	0	0	0	0	0	0	0	0	0	0	0	0	0	0	0	0	0
Unaffiliated Eubacteriales	0	0	0.04	0	0	0	0	0	0	0	0	0	0	0	0	0	0	0	0
Unaffiliated EV818SWSAP88	0	0	0	0	0	0	0	0	0	3.48	0	0	0	0	0	0	0	0	0
Unaffiliated Fibrobacterales	0.05	0	0	0	0	0	0	0	0	0	0	0	0	0	0	0	0	0	0
Unaffiliated Gammaproteobacteria	1.03	0.91	0.55	0.03	0	0	1.04	2.54	0.27	0	0	0	0	2.97	0.60	0	2.93	0.04	0.25
Unaffiliated Gracilibacteria	0.32	0	0.03	0	0	0	0	0	0	0	0	0	0	0	0	0	0	0	0
Unaffiliated GWA2-38-13b	0	0	0	0	0	0	0	0	0	0	0	0	0	0	0	0	0	0	0.45
Unaffiliated Halanaerobiales	0	0	0.02	0	0	0	0	0	0	0	0	0	0	0	0	0	0	0	0
Unaffiliated Hyphomicrobiales	0.01	0.65	0	0	0	0	0.32	0.03	1.25	0	0	0	0	0	0.16	0	0	0	1.32
Unaffiliated Hyphomonadales	0.01	0	0	0	0	0	0	0	0	0	0	0	0	0	0	0	0	0	0
Unaffiliated Ignavibacteriales	0	0	0.04	0	0	0	0	0	0	0	0	0	0	0	0	0	0	0	0
Unaffiliated Kapabacteriales	0.04	0.37	0.16	0	0	0	0	0.13	0	0	0	0	0	0	0	0	0	0	0.41
Unaffiliated Latescibacterota	0	0	0.12	0	0	0	0	0	0	0	0	0	0	0	0	0	0	0	0
Unaffiliated Margulisbacteria	0	0	0.00	0	0	0	0	0	0	0	0	0	0	0	0	0	0	0	0
Unaffiliated Marinimicrobia (SAR406 clade)	0.10	0	0.02	0	0	0	0	0	0	0	0	0	0	0	0	0	0	0	0
Unaffiliated MAT-CR-H4-C10	0	0	0.09	0	0	0	0	0	0	0	0	0	0	0	0	0	0	0	0
Unaffiliated Micavibrionales	0	0	0	0	0	0	0	0	0	0	0	0	0	0	0	0	0	0	0.01
Unaffiliated ML602M-17	0	0.71	0.16	0	0	0.29	0	0.36	0.10	0	0	0	0	0	0	0	0	0	0
Unaffiliated MVP-16	0	0	0.04	0	0	0	0	0	0	0	0	0	0	0	0	0	0	0	0
Unaffiliated Oceanospirillales	0	0.27	0	0.77	0	0	0	0	0	0	0	0	0	0	0	0	0	0	0
Unaffiliated OM191	0	0	0.07	0	0	0	0	0	0	0	0	0	0	0	0	0	0	0	0
Unaffiliated OPB41	1.16	0	0	0	0	0	0	0	0	0	0	0	0	0	0	0	0	0	0
Unaffiliated Parcubacteria	7.73	0	0.11	0	0	0	0.42	0.13	0	0	0	0	0	0	0	0	0	0	1.45
Unaffiliated Patescibacteria	0.07	1.20	0	0.22	0	0	0	0	0	0	0	0	0	0	0	0	0	0	0
Unaffiliated PAUC26f	0.08	0	0	0	0	0	0	0	0	0	0	0	0	0	0	0	0	0	0
Unaffiliated PAUC43f marine benthic group	3.48	0	0.15	0	0	0	0	0	0	0	0	0	0	0	0	0	0	0	0
Unaffiliated PeM15	0	0	0	0	0	0	0	0.80	0	0	0	0	0	0	0	0	0	0	0
Unaffiliated Phormidesmiales	0	0	0.11	0	0	0	0	0	0	0	0	0	0	0	0	0	0	0	0
Unaffiliated Pla1 lineage	0	0	0.03	0	0	0	0	0	0	0	0	0	0	0	0	0	0	0	0
Unaffiliated Pla4 lineage	0	0	0.05	0.05	0	0	0	0	0	0	0	0	0	0	0	0	0	0	0
Unaffiliated Planctomycetales	0	0	0	0	0	0	0	0	0	0	0	0	0	0.46	0	0	0	0	0
Unaffiliated Planctomycetota	0.07	0	0	0.00	0	0.03	0	0	0	0	0	0	0	0	0	0	0.02	0	0

Unaffiliated Pseudomonadales	0.01	0.33	0.37	0.17	0	0	0	0	0	0	0	0	0	0	0	0	0	2.10	0
Unaffiliated Puniceispirillales	0.74	0	0.11	0	0	0	0	0	0	0	0	0	0	0	0	0	0	0	0
Unaffiliated RBG-13-54-9	0	0	0.03	0	0	0	0	0	0	0	0	0	0	0	0	0	0	0	0
Unaffiliated Rhizobiales	0.01	0	0	0	0	0	0	0	0	0	0	0	0	0	0	0	0	0	0
Unaffiliated Rhodospirillales	0	0	0.42	0.97	0	0	0.04	0.07	0	0	0	0	0	0	0	0	0	0	0.07
Unaffiliated Rhodothermia	0	0	0	0.81	0	0	0	0	0	0	0	0.05	0	0	0	0	0	0	0
Unaffiliated Rickettsiales	0	0	0	0.04	0	0	0	0	0	0	0	0	0	0	0	0	0	0	0
Unaffiliated Rs-M47	0.04	0	0	0	0	0	0	0	0	0	0	0	0	0	0	0	0	0	0
Unaffiliated SAR324	0	0	0	0	0	0	0	0.66	0	0	0	0	0	0	0	0	0	0	0
Unaffiliated SBR1031	0.15	0	0.09	0	0	0	0	0	0	0	0	0	0	0	0	0	0	0	0
Unaffiliated Sericytochromatia	0.03	0	0	0	0	0	0	0.02	0	0	0	0	0	0	0	0	0	0	0
Unaffiliated SM1A07	2.80	0.12	0	0	1.00	0	0	0	0	0	0	0	0	0	0	0	0	0	3.45
Unaffiliated Sphingobacteriales	0.09	0	0	0	0.02	0	0	0.30	0	0	0	0	0	0	0	0	0	0	0
Unaffiliated SS1-B-02-17	0.18	0	0	0	0	0	0	0	0	0	0	0	0	0	0	0	0	0	0
Unaffiliated Subgroup 21	0	0	0.21	0	0	0	0	0	0	0	0	0	0	0	0	0	0	0	0
Unaffiliated Sumerlaeia	0	0	0.27	0	0	0	0	0	0	0	0	0	0	0	0	0	0	0	0
Unaffiliated Thalassobaculales	0	0	0.04	0	0	0	0	0.10	0	0	0	0	0	0	0	0	0	0	0
Unaffiliated Thermovenabulales	0.40	0.60	5.09	16.59	2.80	0	0	0	0.38	0	0	0	0	0	0	0	3.77	8.19	0
Unaffiliated Thermovenabulia	0	0.48	4.70	0.40	0	0	0	0	0	0	0	0	0	0	0	0	0	0	0
Unaffiliated Thiohalobacterales	0	0	0.04	0	0	0	0	0	0	0	0	0	0	0	0	0	0	0	0
Unaffiliated Thiohalomonadales	0	0	0	0	0	0.36	0	0	0	0	0	0	0	0	0	0	0	0	0
Unaffiliated vadinHA49	0	0	0.17	0	0	0	0	0	0	0	0	0	0	0	0	0	0	0	0
Unaffiliated Vampirovibrionales	0.07	0	0.07	0.96	0	0	0	0	0	0	0	0	0	0	0	0	0	0	0
Unaffiliated WCHB1-41	0	0	0.08	0	0	0	0	0	0	0	0	0	0	0	0	0	0	0	0

Table S2. Archaeal genera with ambiguous affiliations and relative abundances <1.0 %

Genus	VS7	VS5	VS2	VS9 sed	VS1new	HL345	VS1	VS4	VS6	HL450	VS3	VS8	VFS	VCR	STP	CP	SM16	SM19	SSR
Halorubrum	0	38.68	0	23.95	0.80	28.12	33.14	34.53	51.24	30.70	55.06	20.95	56.76	12.09	32.80	89.66	14.09	19.53	55.75
Halomarina	0	3.68	0	0	1.37	0	7.11	30.91	4.44	12.96	0	0	7.47	39.50	4.29	0	3.84	13.05	0
Haloplanus	0	5.54	0	10.93	14.83	0	15.55	7.03	15.19	0	0	0	11.34	2.51	6.06	0.23	4.14	3.35	0
Halobacterium	0	0	0	0	3.53	48.35	0	0	0	0	0	0	0	2.48	3.27	0	0.82	29.58	0
Halodesulfurarchaeum	0	0	63.08	0	0	0	0	0	0	0	0	0	0	0	0.41	0	0	0	23.45

Nanosalinicola	68.57	0	0	1.40	0	0	0	0	0	2.80	0	1.95	0	2.04	1.30	0	4.18	0	0
Halobellus	0	0	0	15.76	18.22	0	3.24	0.44	1.29	12.96	0	10.16	3.28	2.08	7.61	0	1.88	0	0
Unc. Haloferacaceae	14.07	15.37	6.81	2.96	4.73	2.65	0.12	0	0	2.29	0	0.68	0.91	3.61	3.04	0.79	6.78	4.00	0
Halobaculum	0	1.95	0	0	0	0	9.99	9.30	3.20	0	15.48	6.88	15.74	1.88	0.69	0	0	0	0
Natronomonas	0	0	8.58	8.48	0	3.25	0	0	0	3.51	8.09	1.45	0	0	2.46	0	28.07	0	0
Haloarcula	0	0	0	3.15	0	0	3.20	3.48	12.03	6.43	0	18.37	2.23	0	0.41	0	0	0	0
Halogeometricum	0	0	0	0	0	0	0	0	0	0	0	35.87	0	0	0	0	0	0	0
Halapricum	0	0	0	3.49	0	0	0.25	0.02	0.85	0	0	0	0	4.72	1.85	1.90	12.57	9.30	0
Unc. SCGC AAA011-D5	7.47	2.71	0	0.29	0	0	0	0	0	0	0	0	0	0	0	0	0.21	0	20.80
Haloredivivus	0	9.05	0	0	4.31	0	7.07	3.01	3.26	0	0	0	1.08	0	1.61	1.07	0	0	0
Nanohalobium	0	0	0	6.34	0	1.11	1.13	0.35	0.75	9.97	0	0	0	4.33	3.15	2.78	0	0	0
Nanosalina	0	5.77	0	0	0	0	4.52	2.83	0.20	2.68	0	0	0	0	5.21	0	1.55	0	0
Nanopetraeus	0	0	0	1.16	0	0	0	0	0	9.57	0	0	0	6.20	1.96	0	0.95	0	0
Halorientalis	0	0	0	2.68	0	0	0.18	0	0	0	0	0	0	12.68	2.04	0	0.30	0	0
Halovenus	0	0	0	0	0	12.43	0	0	0	0	0	0	0	0	1.54	0	0.89	0	0
Nanohalococcus	0	0	0	0	0	0	6.67	2.75	0.83	0	0.97	3.17	0	0	0	0	0	0	0
Halogranum	0	0	0	0	0	0	0	0	0.41	0	13.74	0	0	0	0	0	0	0	0
Salinirubrum	0	0	3.27	0	0	0	4.35	0.98	4.06	0	0.13	0	0.99	0	0	0	0	0	0
Nanohaloarchaeum	0	0	0	1.95	1.10	0	1.40	0	0	0	0	0	0	0	1.95	1.23	2.87	1.67	0
Haloquadratum	0	0	0	8.84	0	0	0	0	0	0	0	0	0	0	0.97	1.00	0	0	0
Halococcus	0	0	0	0	0	0	0	0	0	0	0	0	0	0	5.27	0	0	5.23	0
Halonotius	0	0	0	1.26	0	0	0	0	0	0	0	0	0	0	6.97	0	0	0	0
Nanohalovita	0	0	0	0	0	0	1.35	0.66	1.50	0	0	0	0.21	1.47	2.02	0.84	0	0	0
Haloarchaeobius	0	0	0	0	0	0	0	0	0	0	0	0	0	0	0	0	6.30	1.09	0
Halomicrobium	0	0	0	1.64	0	0.15	0	0.77	0	0	0	0	0	0	1.15	0	0	3.57	0
Unc. Haloarculaceae	0	0	0	1.77	0	1.74	0	0	0	0	0	0	0	0	0.65	0	0	2.09	0
Halomicroarcula	0	0	0	0.47	0	0	0	2.67	0	0.40	0	0.43	0	2.23	0	0	0	0	0
Natronoarchaeum	0	0	0	0	0	0	0	0	0	5.73	0	0	0	0.32	0	0	0	0	0
Haloparvum	0	0	0	0	0	0	0	0	0	0	0	0	0	0	0	0	2.45	0	0
Halosimplex	0	0	0	0	2.29	0	0	0	0	0	0	0	0	0	0	0	0	0	0
Unc. Halomicrobiaceae	0	0	0	0	0	0.48	0.30	0	0.49	0	0	0.10	0	0.91	0.01	0	0	0	0
Haloferax	0	0	0	2.07	0	0	0	0	0	0	0	0	0	0	0	0	0	0	0
Haloterrigena	0	0	0	0	0	1.56	0	0	0	0	0	0	0	0	0	0	0	0	0
Methanohalobium	0	0	1.36	0	0	0	0	0	0	0	0	0	0	0	0	0	0	0	0

Halorubellus	0	0	0	0	0	0	0	0	0	0	0	0	0	0	0	0	1.34	0	0
Unc. Halobacteriaceae	0	0	0	0	0	0.15	0	0	0	0	0	0	0	0.95	0	0	0	0	0
Unc. GW2011_GWC1_47_15	0	0.87	0	0	0	0	0	0	0	0	0	0	0	0	0	0	0	0	0
Salinarchaeum	0	0	0	0	0	0	0	0	0	0	0	0	0	0	0	0.50	0	0	0
Unc. Halorubraceae	0	0	0	0	0	0	0	0	0	0	0	0	0	0	0	0	0	0.08	0
Unc. J07HX5	0	0	0	0	0	0	0	0	0	0	0	0	0	0	0.02	0	0	0	0
Unaffiliated Nanohaloarchaeota	9.89	0	0	0	0	0	0	0	0	0	0	0	0	0	0	0	0	0	0
Unaffiliated Halobacterales	0	0	0	0	0	0	0	0	0	0	0	0	0	0	0.09	0	1.34	7.44	0
Unaffiliated Halobacterota	0	0	0	1.21	0	0	0	0	0	0	0	0	0	0	0	0	0	0	0
Unaffiliated Thermoplasmata	0	0	11.72	0.20	0	0	0	0	0	0	0	0	0	0	0	0	0	0	0
Unaffiliated Woesearchaeales	0	16.37	5.18	0	0	0	0.44	0.29	0.26	0	6.54	0	0	0	1.20	0	0	0	0

Table S3. Bacterial genera with relative abundances >1.0 %

Genus	VS7	VS5	VS2	VS9 sed	VS1new	HL345	VS1	VS4	VS6	HL450	VS3	VS8	VFS	VCR	STP	CP	SM16	SM19	SSR
Salinibacter	0.12	0	0.04	23.41	44.49	0.41	3.43	0.53	2.31	4.01	0	90.39	0.61	24.82	69.71	6.77	57.02	40.35	10.98
Salisaeta	0.43	2.84	0.35	0.79	18.35	0	13.83	3.36	51.36	0	12.53	0.22	15.53	4.76	2.24	28.99	0	0.38	19.21
Dactylococcopsis PCC-8305	0	3.16	0.10	0.12	0.43	0	9.02	3.71	12.47	0	13.42	0	11.50	0	0	15.23	0	0	5.32
Achromobacter	0.01	0	0	0	0	0	12.39	1.60	0	0	16.44	0	35.07	7.44	0.02	0	0	0	0
Unc. Bradymonadaceae	1.35	1.66	0.79	1.96	11.09	0	0	0.17	0	0	0	0	0	0	4.62	0	5.13	24.53	15.23
Halovibrio	0	0.21	0	0.80	0	39.90	0	0	0	21.39	0	0	0	0	0	0	2.39	0	0
Puniceicoccus	0.07	12.97	0.16	0.01	1.55	0	9.03	12.52	2.02	0	0	0	0	1.02	0.60	3.14	0	0	0
Halanaerobium	2.58	8.27	9.40	6.15	0	0.14	0.01	0	0.35	1.07	0	0	0	2.69	0.56	0	0	0	7.65
Unc. Halobacteroidaceae	0.02	0.17	0.23	0.92	1.19	0	0	0.18	0	0	0	0	0	0	0	35.11	0	0	0
Stenotrophomonas	0	0	0	0	0	0	8.48	1.18	0.03	0	5.66	0	15.08	6.20	0	0	0	0	0
Aquabacterium	0	0	0	0	0	0	1.69	0	0	0	7.78	0	18.90	5.52	0	0	0	0	0
Halomonas	0	0.94	0.18	0	0	16.29	0	0	0	6.28	0.43	0	0	2.35	0	0	0	0	0
Idiomarina	0.02	1.54	0.38	0	0	8.59	0	0	0.47	11.76	0.64	0	0	0	0	0	0	0	0
Rhodovibrio	1.50	0.09	0.88	3.16	2.01	1.38	2.05	0.24	10.02	0	0	0	0	0	0.67	0	0	0	0.45
Coraliomargarita	0	15.39	0.18	0	0	0	4.26	1.14	0.97	0	0	0	0	0.34	0	0	0	0	0
Aliifodinibius	2.53	0.67	1.17	0	0	7.30	0	0	0	6.15	0	1.51	0	0	0	0	0	2.72	0
Unc. Balneolaceae	2.96	1.35	0.35	0.01	0	0	0.33	1.17	1.50	0	8.85	1.16	0	0	0	0	1.31	1.77	0
Salinivibrio	0.10	0	0	0	0	0	0	0	0	0	19.58	0	0	0	0	0	0	0	0.44
Unc. Halomonadaceae	0.28	2.13	0.28	2.70	0	4.42	0	0	0	6.55	0	0	0	0	0	0	1.15	0.79	0.76
Psychrobacter	0	0	0	0	0	0	0	0	0	0	0	0	0	17.50	0	0	0	0	0
Unc. Phycisphaeraceae	6.45	0	0.58	2.10	0	0	0.02	0	0	0	0	0	0	0	0	0	6.41	0.24	1.23
Halofilum	0.46	1.04	0.20	0	0	6.04	0	0	0	6.55	0	0	0	0	0	0	1.42	0.65	0
Longimonas	0	0.81	0.13	0	1.49	0	1.02	0.08	4.86	0	3.20	0	2.34	0	0	0	0	0	2.28
Unc. Micavibrionaceae	0.25	0	0	0.42	1.14	3.77	0.88	2.01	0.69	5.88	0	0	0	0	0	0.19	0	0	0
Desulfovermiculus	0.57	2.10	3.55	6.39	0	0	0	0	0	0	0	0	0	0	0.31	0	1.32	0	0.73
Thiohalorhabdus	6.87	0.19	5.54	0.34	0	0	0	0	0	0	0	0	0	0	0	0	0.65	0	0
Psychroflexus	0	0.76	0.08	0	0.15	0	0.06	0	0	0	0	0	0	2.00	0.38	0	0	0	7.98
Truepera	3.08	2.37	0.40	0	0.62	0	0.94	2.06	0.83	0	0.37	0	0	0	0	0	0	0	0
Halopeptonella	0	1.12	0.42	0	1.00	2.09	0	3.02	0	0	0	0	0	2.89	0	0	0	0	0
Unc. E6aC02	0.54	1.03	1.39	1.28	0	0	0	0	0	0	0	0	0	0	0	0	0	0	5.28
Lentimonas	0	0.45	0.09	0.18	0	0	1.31	7.30	0	0	0	0	0	0	0	0	0	0	0

Unc. Marinilabiliaceae	2.47	0.58	3.62	0.39	0	0	0	0	0.21	0	0	0	0	0	0	0	0	0	1.97
Roseovarius	0	0	0	0	0	0	0	0	0	8.42	0	0	0	0	0.39	0	0	0	0
Unc. Rhodobacteraceae	2.36	0.02	0.25	0.21	0.01	0	2.81	2.99	0	0	0	0	0	0	0	0	0	0	0
Unc. Saprospiraceae	0.43	1.48	0.13	0	0	0	0.99	5.49	0.08	0	0	0	0	0	0	0	0	0	0
Roseivivax	0	0	0.65	0	2.12	0	1.11	2.16	0.27	0	0	0	0	0	1.13	0	0	0	0
WDS1C4	0	0	0	0	0.30	0	0	6.94	0	0	0	0	0	0	0	0	0	0	0
Verruc-01	0.17	0.33	1.13	0	0	0	1.80	1.21	1.03	0	0	0	0	0	0	0	0	0	1.44
Marinobacter	0.98	1.45	1.32	0	0	3.22	0	0	0.12	0	0	0	0	0	0	0	0	0	0
Halanaerobacter	0	0.25	0.16	1.29	0	0	0	0	0	0	0	0	0	0	0	4.73	0	0	0.55
Fusobacterium	0	0	0	0	0	0	0	0	0	0	0	0	0	6.01	0	0	0	0	0
Spirochaeta 2	0.56	0	1.77	0	0	0	0.10	0	0.10	0	0	0	0	0	0	0	0	0	3.12
Oceanicaulis	0.39	0	0.48	0	0.40	0	0.16	3.27	0.36	0	0.27	0	0	0	0.27	0	0	0	0
Gracilimonas	0.13	0.48	0.23	0	0.01	0	0.44	0.42	0	0	3.89	0	0	0	0	0	0	0	0
Candidatus *Aquiluna*	0	3.36	0.12	0	0	0	0	1.69	0	0	0.22	0	0	0	0	0	0	0	0
Unc. C2U	1.43	0	0	0	0.41	0	1.79	0.59	0	0	0	0.51	0	0	0.60	0	0	0	0
Unc. Lenti-02	0	0.41	3.58	0.79	0	0	0.27	0.03	0	0	0	0	0	0	0	0	0	0	0
Maritimibacter	0	0	0	0	0	0	0	0	0	4.95	0	0	0	0	0	0	0	0	0
Halospina	0.57	2.00	0	0.17	0	0	0	0	0.38	0	0	0	0	0	0	0	0.46	0.22	0.99
Sphingomicrobium	0	0	0	0	0	0	0	0	0	4.68	0	0	0	0	0	0	0	0	0
Desulfosalsimonas	0.30	0	3.47	0.68	0	0	0	0	0	0	0	0	0	0	0	0	0	0	0
MAT-CR-P4-C12	0.10	0	0.24	0.86	0.04	0	0	0	0	0	0	0	0	0.05	0	0	0.83	2.17	0
Rhodopirellula	0.27	0.41	0.02	0	0	0	2.40	1.06	0	0	0	0	0	0	0.02	0	0	0	0
Limibaculum	0.90	0	0.79	0	0.68	0	0	1.81	0	0	0	0	0	0	0	0	0	0	0
Roseicyclus	0.35	0	0.23	0	0	0	1.22	2.14	0	0	0	0	0	0	0.11	0	0	0	0
Anaerobiospirillum	0	0	0	0	0	0	0	0	0	0	0	0	0	3.78	0	0	0	0	0
Guyparkeria	0	0.53	3.06	0	0	0	0.02	0	0	0	0	0	0	0	0	0	0	0	0
Wenzhouxiangella	2.81	0	0.34	0	0.41	0	0	0	0	0	0	0	0	0	0	0	0	0	0
Plesiomonas	0	0	0	0	0	0	0	0	0	0	0	0	0	3.53	0	0	0	0	0
Unc. Alcanivoracaceae	0.09	0	0	0.73	0	0	0	0	0	0	0	0	0	0	0	0	1.02	1.66	0
Peptoclostridium	0	0	0	0	0	0	0	0	0	0	0	0	0	3.40	0	0	0	0	0
DS001	0	2.64	0	0	0	0	0	0.54	0	0	0	0	0	0	0	0	0	0	0
Salinispirillum	0	1.07	0.03	0	0	1.97	0	0	0	0	0	0	0	0	0	0	0	0	0
Halobacillus	0	0	0	0	0	0	0	0	0	0	0	3.01	0	0	0	0	0	0	0

Albimonas	0.57	0	0.06	0	0	0	2.03	0.13	0.21	0	0	0	0	0	0	0	0	0	0
Unc. Hyphomonadaceae	0.13	0	0.23	0	0.32	0	1.54	0.49	0.20	0	0	0	0	0	0	0	0	0	0
SM1A02	0	0	0.16	0	0	0	0	0.04	0	0	0	0	0	0	0	2.70	0	0	0
Catalinimonas	0	0	0.12	0	0	0	0.20	0.21	0.68	0	1.64	0	0	0	0	0	0	0	0
Fusibacter	0.27	0.97	0.43	1.12	0	0	0	0	0	0	0	0	0	0	0	0	0	0	0
Unc. Rhodothermaceae	0.39	0	0	0.70	0.26	0	0.07	0	0	0	0.58	0	0	0	0	0	0	0.74	0
Pseudohongiella	0	1.44	0.15	0.46	0	0	0	0.17	0	0	0	0	0	0	0	0	0	0.33	0
Halanaerobaculum	0.01	0	0	0.01	0	0	0	0	0	0	0	0	0	0	0.06	2.45	0	0	0
Parvularcula	0	0	0	0	0.03	0	0.43	0.05	2.00	0	0	0	0	0	0	0	0	0	0
Owenweeksia	0.27	1.39	0.28	0.06	0	0	0	0.19	0.05	0	0	0	0	0	0	0	0	0	0.19
Legionella	0.49	0	0	0	0	0	0	0.60	0	1.34	0	0	0	0	0	0	0	0	0
Unc. Comamonadaceae	0	0	0	0	0	0	0	0	0	2.41	0	0	0	0	0	0	0	0	0
Desulfohalobium	0.17	0.26	0	1.95	0	0	0	0	0	0	0	0	0	0	0	0	0	0	0
Thiohalospira	0.42	0.66	0.29	0	0	0	0	0	0	0	0	0	0	0	0	0	0	0	0.93
Alcanivorax	0.05	0	0.46	1.68	0	0	0	0	0	0	0	0	0	0	0	0	0	0	0
Limimonas	0	0	0	0.34	0.59	0	0	0	0	0	0	0	0	0	1.23	0	0	0	0
Unc. Desulfosarcinaceae	0.01	0	1.64	0.00	0	0	0	0	0	0	0	0	0	0	0	0	0	0	0.32
Acinetobacter	0	0	0	0	0	0	0	0	0.93	0	0.64	0.42	0	0	0	0	0	0	0
Unc. Desulfobacteraceae	0.25	0.95	0	0.77	0	0	0	0	0	0	0	0	0	0	0	0	0	0	0
Cyanobium PCC-6307	0	0	0.48	0	0	0	0	0	0	1.47	0	0	0	0	0	0	0	0	0
Peredibacter	0.27	0.79	0.37	0	0	0	0	0.48	0	0	0	0	0	0	0	0	0	0	0
Qipengyuania	0	0	0	0	0	0	1.11	0.69	0	0	0	0	0	0	0	0	0	0	0
GKS98 freshwater group	0	0.15	0.04	0	0	0.65	0.39	0.50	0	0	0	0	0	0	0	0	0	0	0
Pontimonas	0	1.36	0	0	0	0	0	0	0.30	0	0	0	0	0	0	0	0	0	0
Methyloversatilis	0	0	0	0	0	0	0	0	0	0	1.64	0	0	0	0	0	0	0	0
Flexistipes	0.28	0	1.16	0.19	0	0	0	0	0	0	0	0	0	0	0	0	0	0	0
Silicimonas	1.58	0	0.04	0	0	0	0	0	0	0	0	0	0	0	0	0	0	0	0
Cnuella	0	0	0	0	0	0	0	0	0	1.60	0	0	0	0	0	0	0	0	0
Unc. Desulfobulbaceae	1.34	0	0.24	0	0	0	0.02	0	0	0	0	0	0	0	0	0	0	0	0
Sediminimonas	1.59	0	0	0	0	0	0	0	0	0	0	0	0	0	0	0	0	0	0
Unc. Bacillaceae	0	0.34	0	0	0	0	0	0.11	0	0	0	1.07	0	0	0	0	0	0	0
Unc. Ectothiorhodospiraceae	0	0	0.09	0	0	0	0	0.23	0	0	0	0	0	0	0	0	0.57	0.61	0
Phormidium MBIC10003	0	0	0	0	0	0	0	0	0	0	1.48	0	0	0	0	0	0	0	0

Unc. Methylomonadaceae	0	0	0	0	0	0	0	0	0	1.47	0	0	0	0	0	0	0	0	0
Halospirulina CCC Baja-95 Cl.2	0.01	0	1.21	0	0	0	0	0.01	0.22	0	0	0	0	0	0	0	0	0	0
Tangfeifania	0	0	1.39	0.01	0	0	0	0	0	0	0	0	0	0	0	0	0	0	0
Halodurantibacterium	0	0	0	0	0	0	0	0	0	0	0	0	0	0	1.38	0	0	0	0
Candidatus *Endoecteinascidia*	0	0	0	0	0	0	0	1.36	0	0	0	0	0	0	0	0	0	0	0
Unc. Cyclobacteriaceae	0.47	0.15	0.10	0	0	0	0	0	0	0	0	0	0	0	0	0	0	0	0.61
ADurb.Bin120	1.05	0	0.26	0	0	0	0	0	0	0	0	0	0	0	0	0	0	0	0
Sediminispirochaeta	0	0	1.29	0	0	0	0	0	0	0	0	0	0	0	0	0	0	0	0
Bacteroides	0	0	0	0	0	0	0	0	0	0	0	0	0	1.28	0	0	0	0	0
Sumerlaea	0	0	0.78	0	0	0	0	0	0	0	0	0	0	0	0	0	0	0	0.49
Litoricola	0	0.36	0	0	0	0	0.16	0.59	0.15	0	0	0	0	0	0	0	0	0	0
Methylosphaera	0	0	0	0	0	1.23	0	0	0	0	0	0	0	0	0	0	0	0	0
Anaerophaga	0.21	0.16	0.86	0	0	0	0	0	0	0	0	0	0	0	0	0	0	0	0
Phycisphaera	0	0	0	0	0	0	0.56	0.63	0	0	0	0	0	0	0	0	0	0	0
Geotoga	0	0.65	0.52	0	0	0	0	0	0	0	0	0	0	0	0	0	0	0	0
Izimaplasma	0	0.53	0.13	0	0	0.09	0	0.33	0	0	0	0	0	0	0.09	0	0	0	0
Geitlerinema PCC-7105	0	0	1.14	0	0	0	0	0	0	0	0	0	0	0	0	0	0	0	0
Rhodobaculum	0.27	0	0	0	0	0	0.85	0	0	0	0	0	0	0	0	0	0	0	0
Rhodovulum	0	0	0.06	0	0	0	0.68	0.31	0.07	0	0	0	0	0	0	0	0	0	0
Arsenophonus	0	0	0	0	1.11	0	0	0	0	0	0	0	0	0	0	0	0	0	0
Candidatus *Berkiella*	0.96	0	0	0	0	0	0	0.15	0	0	0	0	0	0	0	0	0	0	0
Aquisalimonas	0	0	0	0	0	1.10	0	0	0	0	0	0	0	0	0	0	0	0	0
Coxiella	0	0	0	0	0	0	0	0.40	0	0	0	0	0	0	0	0	0.67	0	0
Ferrimonas	0	0	0	1.05	0	0	0	0	0	0	0	0	0	0	0	0	0	0	0
Arhodomonas	1.05	0	0	0	0	0	0	0	0	0	0	0	0	0	0	0	0	0	0
Desulfotignum	0	0	1.01	0	0	0	0	0	0	0	0	0	0	0	0	0	0	0	0
HIMB11	0	0	0.03	0	0	0	0.27	0.71	0	0	0	0	0	0	0	0	0	0	0
Not affiliated	28.83	11.77	19.11	30.52	9.37	1.03	6.34	10.97	3.61	3.48	0	1.72	0	4.43	14.99	0	19.66	22.06	10.71
Other	14.55	1.77	17.41	5.28	0.45	0.41	3.50	10.01	1.14	0.53	0.75	0.00	0.96	0.00	0.60	0.68	0.00	0.78	1.13

Table S4. Archaeal genera with relative abundances >1.0 %

Genus	VS7	VS5	VS2	VS9 sed	VS1new	HL345	VS1	VS4	VS6	HL450	VS3	VS8	VFS	VCR	STP	CP	SM16	SM19	SSR
Halorubrum	0	38.68	0	23.95	0.80	28.12	33.14	34.53	51.24	30.70	55.06	20.95	56.76	12.09	32.80	89.66	14.09	19.53	55.75
Halomarina	0	3.68	0	0	1.37	0	7.11	30.91	4.44	12.96	0	0	7.47	39.50	4.29	0	3.84	13.05	0
Haloplanus	0	5.54	0	10.93	14.83	0	15.55	7.03	15.19	0	0	0	11.34	2.51	6.06	0.23	4.14	3.35	0
Halobacterium	0	0	0	0	3.53	48.35	0	0	0	0	0	0	0	2.48	3.27	0	0.82	29.58	0
Halodesulfurarchaeum	0	0	63.08	0	0	0	0	0	0	0	0	0	0	0	0.41	0	0	0	23.45
Nanosalinicola	68.57	0	0	1.40	0	0	0	0	0	2.80	0	1.95	0	2.04	1.30	0	4.18	0	0
Halobellus	0	0	0	15.76	18.22	0	3.24	0.44	1.29	12.96	0	10.16	3.28	2.08	7.61	0	1.88	0	0
Unc. Haloferacaceae	14.07	15.37	6.81	2.96	4.73	2.65	0.12	0	0	2.29	0	0.68	0.91	3.61	3.04	0.79	6.78	4.00	0
Halobaculum	0	1.95	0	0	0	0	9.99	9.30	3.20	0	15.48	6.88	15.74	1.88	0.69	0	0	0	0
Natronomonas	0	0	8.58	8.48	0	3.25	0	0	0	3.51	8.09	1.45	0	0	2.46	0	28.07	0	0
Haloarcula	0	0	0	3.15	0	0	3.20	3.48	12.03	6.43	0	18.37	2.23	0	0.41	0	0	0	0
Halogeometricum	0	0	0	0	0	0	0	0	0	0	0	35.87	0	0	0	0	0	0	0
Halapricum	0	0	0	3.49	0	0	0.25	0.02	0.85	0	0	0	0	4.72	1.85	1.90	12.57	9.30	0
Unc. SCGC AAA011-D5	7.47	2.71	0	0.29	0	0	0	0	0	0	0	0	0	0	0	0	0.21	0	20.80
Haloredivivus	0	9.05	0	0	4.31	0	7.07	3.01	3.26	0	0	0	1.08	0	1.61	1.07	0	0	0
Nanohalobium	0	0	0	6.34	0	1.11	1.13	0.35	0.75	9.97	0	0	0	4.33	3.15	2.78	0	0	0
Nanosalina	0	5.77	0	0	0	0	4.52	2.83	0.20	2.68	0	0	0	0	5.21	0	1.55	0	0
Nanopetraeus	0	0	0	1.16	0	0	0	0	0	9.57	0	0	0	6.20	1.96	0	0.95	0	0
Halorientalis	0	0	0	2.68	0	0	0.18	0	0	0	0	0	0	12.68	2.04	0	0.30	0	0
Halovenus	0	0	0	0	0	12.43	0	0	0	0	0	0	0	0	1.54	0	0.89	0	0
Nanohalococcus	0	0	0	0	0	0	6.67	2.75	0.83	0	0.97	3.17	0	0	0	0	0	0	0
Halogranum	0	0	0	0	0	0	0	0	0.41	0	13.74	0	0	0	0	0	0	0	0
Salinirubrum	0	0	3.27	0	0	0	4.35	0.98	4.06	0	0.13	0	0.99	0	0	0	0	0	0
Nanohaloarchaeum	0	0	0	1.95	1.10	0	1.40	0	0	0	0	0	0	0	1.95	1.23	2.87	1.67	0
Haloquadratum	0	0	0	8.84	0	0	0	0	0	0	0	0	0	0	0.97	1.00	0	0	0
Halococcus	0	0	0	0	0	0	0	0	0	0	0	0	0	0	5.27	0	0	5.23	0
Halonotius	0	0	0	1.26	0	0	0	0	0	0	0	0	0	0	6.97	0	0	0	0
Nanohalovita	0	0	0	0	0	0	1.35	0.66	1.50	0	0	0	0.21	1.47	2.02	0.84	0	0	0
Haloarchaeobius	0	0	0	0	0	0	0	0	0	0	0	0	0	0	0	0	6.30	1.09	0
Halomicrobium	0	0	0	1.64	0	0.15	0	0.77	0	0	0	0	0	0	1.15	0	0	3.57	0
Unc. Haloarculaceae	0	0	0	1.77	0	1.74	0	0	0	0	0	0	0	0	0.65	0	0	2.09	0
Halomicroarcula	0	0	0	0.47	0	0	0	2.67	0	0.40	0	0.43	0	2.23	0	0	0	0	0

Natronoarchaeum	0	0	0	0	0	0	0	0	0	5.73	0	0	0	0.32	0	0	0	0	0
Haloparvum	0	0	0	0	0	0	0	0	0	0	0	0	0	0	0	0	2.45	0	0
Halosimplex	0	0	0	0	2.29	0	0	0	0	0	0	0	0	0	0	0	0	0	0
Unc. Halomicrobiaceae	0	0	0	0	0	0.48	0.30	0	0.49	0	0	0.10	0	0.91	0.01	0	0	0	0
Haloferax	0	0	0	2.07	0	0	0	0	0	0	0	0	0	0	0	0	0	0	0
Haloterrigena	0	0	0	0	0	1.56	0	0	0	0	0	0	0	0	0	0	0	0	0
Methanohalobium	0	0	1.36	0	0	0	0	0	0	0	0	0	0	0	0	0	0	0	0
Halorubellus	0	0	0	0	0	0	0	0	0	0	0	0	0	0	0	0	1.34	0	0
Unc. Halobacteriaceae	0	0	0	0	0	0.15	0	0	0	0	0	0	0	0.95	0	0	0	0	0
Unc. GW2011_GWC1_47_15	0	0.87	0	0	0	0	0	0	0	0	0	0	0	0	0	0	0	0	0
Salinarchaeum	0	0	0	0	0	0	0	0	0	0	0	0	0	0	0	0.50	0	0	0
Unc. Halorubraceae	0	0	0	0	0	0	0	0	0	0	0	0	0	0	0	0	0	0.08	0
Unc. J07HX5	0	0	0	0	0	0	0	0	0	0	0	0	0	0	0.02	0	0	0	0
Unaffiliated Nanohaloarchaeota	9.89	0	0	0	0	0	0	0	0	0	0	0	0	0	0	0	0	0	0
Unaffiliated Halobacterales	0	0	0	0	0	0	0	0	0	0	0	0	0	0	0.09	0	1.34	7.44	0
Unaffiliated Halobacterota	0	0	0	1.21	0	0	0	0	0	0	0	0	0	0	0	0	0	0	0
Unaffiliated Thermoplasmata	0	0	11.72	0.20	0	0	0	0	0	0	0	0	0	0	0	0	0	0	0
Unaffiliated Woesearchaeales	0	16.37	5.18	0	0	0	0.44	0.29	0.26	0	6.54	0	0	0	1.20	0	0	0	0

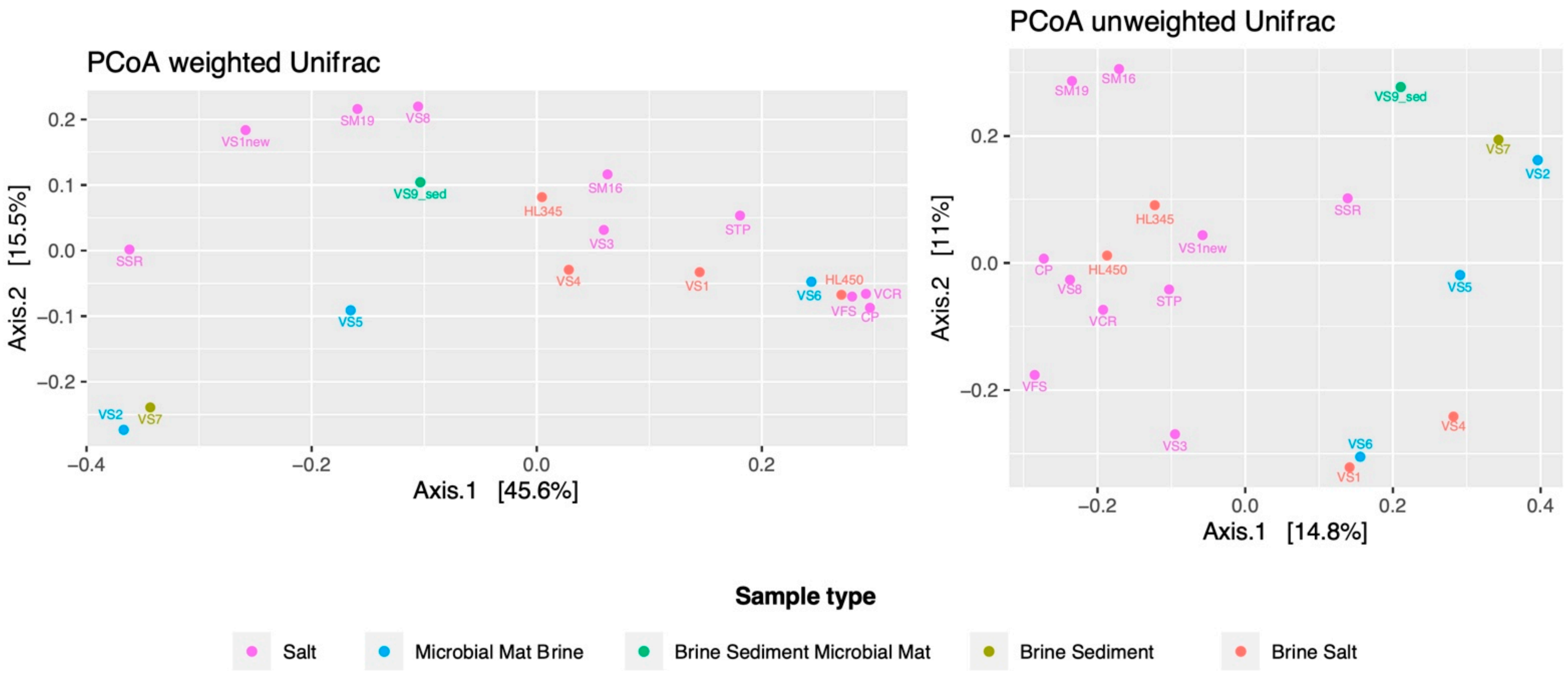

Figure S1. Principal component analysis (PCoA) using unweighted and weighted Unifrac distance calculation for microbial community composition in different solar salterns.

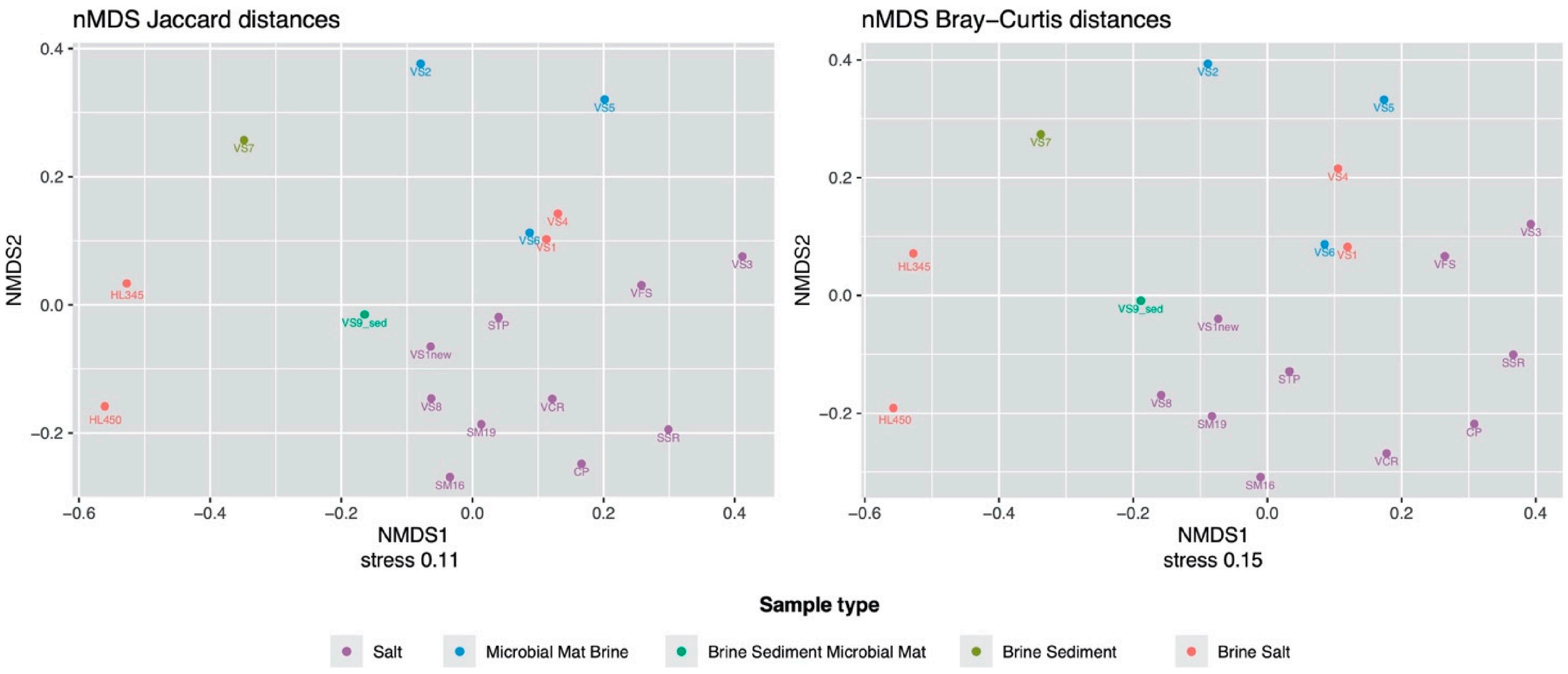

Figure S2. Non-Metric Multi-Dimensional Scaling (NMDS) plots of microbial community composition of analyzed solar salterns and salt samples using Jaccard (left) and Bray–Curtis distances (right).
